# Physical and Dosimetric Optimization of Laser Equipment in Dermatology: A Preliminary Study

**DOI:** 10.1155/2014/151969

**Published:** 2014-09-15

**Authors:** A. Soriani, D. D'Alessio, V. Cattelan, N. Cameli, M. Mariano, S. Ungania, M. Guerrisi, L. Strigari

**Affiliations:** ^1^Medical Physics Laboratory, Regina Elena National Cancer Institute, 00144 Rome, Italy; ^2^Department of Dermatology, San Gallicano Institute, 00144 Rome, Italy; ^3^Department of Physics, Tor Vergata University, 00173 Rome, Italy

## Abstract

The aim of this preliminary study is to investigate the correlation between clinical set-up at present used in the treatment of specific skin conditions and laser beam absorbed power in the tissue. This study focused on the CO_2_ and Nd-Yag laser equipment used in the daily clinical practice in the Department of Dermatology of San Gallicano Institute in Rome. Different types of tissue-equivalent material with various water and haemoglobin concentrations were tested to evaluate laser beam attenuation power. In particular, thinly sliced pork loin, of uniform consistency and without fat, was selected for its high content of haemoglobin to mimic human tissues. An optical power meter was used to measure the power or energy of a laser beam. During measurements, the tissue equivalent phantoms were positioned on the detector head and the laser beam was orthogonally oriented. The results of two experimental set-ups are reported here. The dependence of residual power (W) as a function of *ex vivo* tissue thickness (mm) for different laser output powers was studied. Data were fitted by a parametric logistic equation. These preliminary data allow for more accurately determining the energy fraction released from lasers to the tissues in order to improve clinical outcomes.

## 1. Introduction

Over the last 50 years, lasers have been used to treat a large variety of skin conditions, either inflammatory or neoplastic, in cosmetic and aesthetic dermatology.

These treatments use different laser types classified according to their tissue target and/or tissue interaction. On the basis of this classification, the most commonly used lasers in dermatology are ablative lasers (CO_2_-Erbium), vascular lasers (Nd-Yag, KTP, and PDL), and pigment lasers (Q-switched Nd-Yag, Rubino, and Alexandrite). Through their selective targeting of skin chromophores these lasers have become the treatment of choice for a wide range of cutaneous lesions including angiomas, telangiectasias, lentigo, tattoos, and acne scars and wrinkles [1, 2].

The ever improving design and technology of lasers result in increased safety and efficacy for patients; nonetheless, continuous training and an evolving knowledge are needed. In fact, the penetration of radiation is closely linked to the laser characteristics and to operative conditions. Short-pulse high-peak power (Q-switched or mode-locking) lasers produce high irradiance in a short time, while traditional continuous or long pulsed lasers imply shorter exposures repeated over prolonged time periods. These different mechanisms are likely to produce, on the irradiated tissues, different biological effects that should be taken into consideration to avoid irreversible damage as well as to reduce any potential hazard.

In daily clinical practice, the wavelength alone is perhaps less of a determining factor as far as depth of penetration is concerned—with respect to type of emitter and operating mode, physical design of the applicator, and adopted technique. A working knowledge of tissue targets and light-tissue interaction is essential to use laser appropriately as well as to interpret clinical laser parameters. As a matter of fact, the clinical applications of the laser depend on wavelength peaks of the laser light, pulse durations, and target skin tissue absorption. Furthermore, histological tissue types (muscle, adipose, and bone), lesion location, and skin phototype are critical parameters for determining the effects of laser interaction [3–6].

The aim of this study is to define the correlation between clinical set-up at present used in the treatment of specific skin conditions and laser beam absorbed power in the tissue. An experimental set-up has been performed using different samples simulating human tissues. Measurements of energy-power at different depths in the tissue have been carried out in order to fully understand the effects of tissue-laser interaction for the optimization of clinical outcomes.

It is to be stressed that* ex vivo* tissue used for the tests has rather different characteristics from those of human tissue, being less vascularized and hydrated. At any rate, the information obtained from these experiments can help us better understand the power and energy release mechanisms in irradiated tissues.

## 2. Material and Methods

### 2.1. Lasers

Our attention was devoted to the laser equipment used in the daily clinical practice in the Department of Dermatology of San Gallicano Institute in Rome, namely, traditional CO_2_ laser, and Nd-Yag laser.

Carbon dioxide (CO_2_) lasers were considered for their prominent role and broad spectrum of possible uses in dermatologic surgery. In particular, SmartXideDOT (DEKA M.E.L.A. Calenzano, Italy), in the two modes of laser beam delivery (7′′ handpiece or fractional CO_2_), is utilized in our clinical practice. In this latter mode the system generates perfectly controlled energy pulses (DOTs) by managing the energy per pulse parameter and the DOT spacing between two microscopic wounds (about 0.5 mm). This mode was not analyzed in this preliminary study but will be dealt with in the future. The spot size for the traditional mode, under examination in this study, is 6.5 mm.

Nd-Yag laser (neodymium-doped yttrium aluminum garnet) with a wavelength of 1064 nm long pulsed mode and 5 mm beam diameter was also investigated.

### 2.2. Tissue-Like Phantoms

Different types of tissue-equivalent material with various water and haemoglobin concentrations were used to evaluate laser beam attenuation power. Haemoglobin concentrations are important because of the presence of red chromophores. In particular, beef was selected for its high content of haemoglobin (about 0.1 mg/mL in beef muscle [7]) to mimic well-vascularised tissues (with human muscle-like chromophores); however, due to its lack of uniformity, it proves difficult to repeat the experiments, affecting reproducibility. All the* ex vivo* material tested was sliced and, slice by slice, accurately measured with a caliper in the incident areas. Loin (fat-free pork loin), characterized by high haemoglobin concentration (red chromophores) and low water concentration (about 37% in fat [8] and 58% in dry-cured loin [9]), was selected as test tissue because of its consistency.

### 2.3. Detection System

The detection system (Laser 2000 Ltd, UK) used in this study had two measuring heads (A-2-D12-HCB and A-200-D60-HPB) for the evaluation of the effective power or energy of the incident laser beam. Through conversion of absorbed energy into heat, the thermal head sensor (thermopile) registered the potential differences (*V*_output_) produced by the thermal gradient generated by lasers and proportional to the incident energy or power.

For nonintensive powers, A-2-D12-HCB air cooling system head turned out to be compatible with different laser types allowing high resolution studies for medium power lasers. The power ranges from 1 mW to 2 W, while energy ranges from 0.5 mJ to 2 J. It has a diameter of 12 mm and a spectral range from 0.25 to 11 *μ*m.

The A-200-D60-HPB air forced cooling system head, characterized by a low reflection index, results to be more resistant. The power ranges from 100 mW to 40 W, while energy ranges from 1 to 200 J. It has a diameter of 60 mm and a spectral range varying from 0.25 to 11 *μ*m. However, A-200-D60-HPB head resolution is lower than A-2-D12-HCB. All systems are calibrated (3% accuracy) according to NIST (National Institute of Standards and Technology) and/or PTB (Physikalisch-Technische Bundesanstalt).

The electronic processing system retrieves all necessary data elaborated by the software interface LP-EXPLORER. The software, connected to the head of detection, automatically recognizes the head and, through a control panel, allows the measurements to be selected and made. All data collected and recorded are useful to verify the stability of the signal in CW lasers and any anomalies in PW laser, directly in the GUI (graphical user interface).

### 2.4. Experimental Set-Up

Since in clinical applications laser hand piece is generally located next to the patient skin (about 3–5 mm), during measurements the laser beam was positioned orthogonally to the system head, in contact with the tissue-equivalent phantoms executing continuous circular movements.

**Figure 1:**
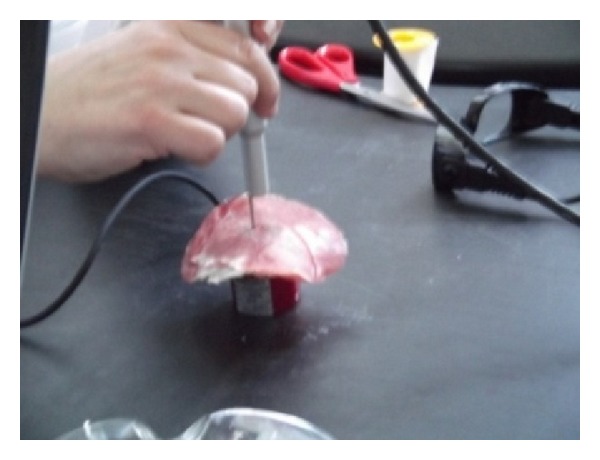
Experimental set-up used for the* ex vivo* phantom and small head measurements.

Power measurements with CO_2_ laser were performed for different thickness (without tissue, 1.6 mm, 3.2 mm, and 4.8 mm). Each set of measurements was repeated with three different powers in CW mode (0.5 W, 0.6 W, 0.8 W). To obtain more accurate incident power detection, a smaller detector head (A-2-D12-HCB) was used (Figure 1).

For CO_2_ laser measurements values of maximum, minimum, and mean power (W), tissue thickness (mm) and residual power (W) were recorded.

**Figure 2:**
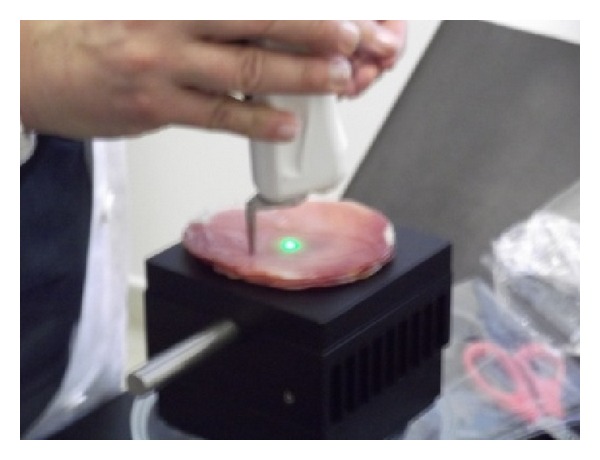
Experimental set-up used for the* ex vivo* phantom and larger head measurements.

Energy measurements with Nd-Yag were performed at five different thicknesses (1.6 mm, 3.2 mm, 4.8 mm, 6.4 mm, and 8.0 mm) with the same phantom (Figure 2). An energy fluence of 125 J/cm^2^ was used. The A-200-D60-HPB head is used in this case. The values of maximum, minimum, and mean power (J), energy fluence (J/cm^2^), tissue thickness (mm), and residual energy (J) were as well recorded.

Laser set-up parameters were chosen, in agreement with clinicians, in order to simulate clinical protocols. The maximum experimental thickness had to be higher than the range (*d*_max⁡_) reducing the residual measured power/energy at a constant value, determined by the measuring head (saturation value).

The net residual energy Δ*E* can be calculated for different tissue thickness as the measured energy value minus the determined saturation value.

For each measuring point a different laser position on the tissue was selected to obtain independent measurements.

### 2.5. Fitting Function

The following empirical function was used to fit the experimental data of power and energy against thickness (*x*):

(1) $$\begin{array}{c} y=\frac{Kae^{-bx}}{1+ae^{-bx}}, \end{array}$$

where *K* is the maximum measured intensity, “*a*” represents a scale factor, and “*b*” determines the curve slope.

The curve was selected being the logistic behaviour representative of the expected physical phenomena.

## 3. Results and Discussion

**Figure 3:**
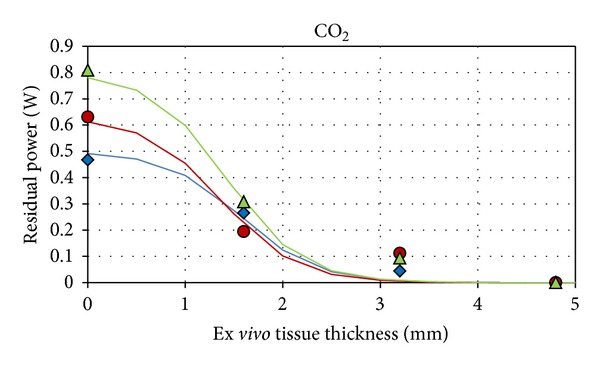
Dependence of the residual power (W) as a function of equivalent-tissue thickness (mm) for three experimental output powers for CO_2_ ((blue diamond) 0.5 W, (red circle) 0.6 W, and (green triangle) 0.8 W).

Figure 3 shows residual power (W) dependence as a function of tissue thickness (mm) for three experimental output powers using CO_2_ laser. A fit with the expected curves is also reported (blue, red, and green lines).

It is to be underscored that for thickness >2 mm the delivered radiation results in being totally absorbed inside the dedicated phantom for all levels of power between the experimental error.

The parameters of each power fitted curve are reported in Table 1.

As regards measurements with the Nd-YAG laser, a certain amount of energy is not absorbed in the first centimeter of tissue and therefore does not contribute to interaction phenomena.

**Figure 4:**
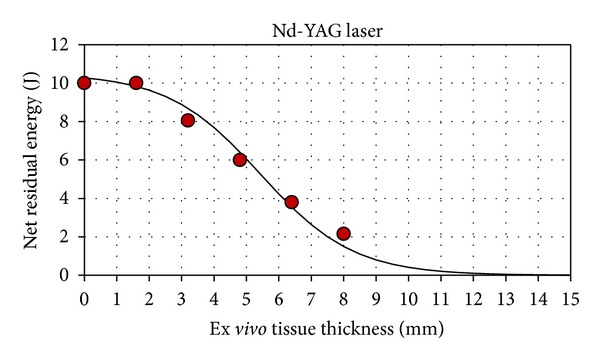
Dependence of the net residual energy Δ*E* (J) as a function of equivalent-tissue thickness (mm) for Nd-Yag ((red circle) 125 J/cm^2^).

Figure 4 represents the dependence of the net residual energy (Δ*E*) in function of the phantom thickness. A fit of experimental data is also shown (black line).

The saturation level is located next to the 15 J energy value. This effect is probably overestimated due to the rather different characteristics of* ex vivo* and human tissue.

It is worth noting that the energy was obtained by converting the selected energy fluence (125 J/cm^2^) with the following expression: 125 × *π* × *r* ^2^, where *r* = 2.5 mm is the radius of laser applicator. The theoretical value of energy thus obtained was 24.5 J while the experimental value was 26.5 J. We could then verify that the average energy value revealed by the power meter head does not differ for more than 10% from that of the set value.

The parameters of the net residual energy fitted curve are reported in Table 1.

Other equivalent tissues—ham or chicken (data not shown)—were tested in order to mimic different biological tissues. Low haemoglobin concentration and high water concentration strongly affected the measurements because of the scatter fraction increasing against depth. As a matter of fact, in such cases the detected power was below the detection system sensitivity level because of the generated high scatter fraction.

Data show that only a small fraction of the incident energy is absorbed by the tissues.

In the CO_2_ laser it is important to establish proper measurement duration and to acquireat least five measurements to check the reproducibility of beam. In this case, the 10.600 nm wavelength emitted as continuous beam destroys tissue by rapidly heating and vaporizing intracellular water. It should however be considered that measurements are affected by an error due to the intrinsic response time of electronics, higher than tissues response. This leads to a general overestimation of values.

As for the potential application of similar studies in the clinical practice, they could be carried out in collaboration with clinicians with limited costs and relevant time-sparing. Our results show that the beam spreading thermal front is one of the source physical parameters characterizing the mechanisms that induce either tissue damage or proper tissue heating, depending on thermal tolerance.

Thin superficial lesions, on the order of hundreds of microns in thickness, receive about the same amount of light throughout their volume due to incident as well as backscattered light reflected from the underlying tissues. This behaviour is a localized effect of laser therapy due to the light administered on the tissue surface, irradiance in (W/cm^2^) or fluence in (J/cm^2^).

In the thick tumour case the aim is to treat the lesion to some desired depth. To treat a volume homogeneously, efficacy ends up to be proportional to the light penetration depth which can be obtained by choosing a proper wave-length. In both cases the knowledge of the beam planar size plays an important role.

In particular, prolonged exposure to moderate (CW) or long pulsed levels of light (PW) produces an absorption effect and relative irreversible local or peripheral tissue damage, depending on the spreading thermal front. These biological effects are different from those associated with the short-pulse high-peak power in which, because of thermal expansion, tissues damage is generated far from the absorbing area. Thus, in the Q-switched lasers, the spreading effect does not occur in the absorbing area.

Moreover, geometrical distortion, the ability to reproduce the real dimensions of the focused beam spots at working distance, is another important parameter. Accuracy out of the acceptable limits of the geometrical distortion affects heterogeneity in power/energy delivery.

## 4. Conclusion

The experimental set-ups analyzed in this study are just a few examples of laser applications in dermatology obtained using standard parameters routinely applied in clinical practice. These preliminary data stress the importance to more accurately know the fraction of released energy from the system to the tissues, to improve the treatment strategy. The main point is the correlation between physical design of the hand piece, the technique used to apply it, wavelength, and laser power in determining clinical outcome. Our team of physicists and physicians will further investigate this relevant topic to improve therapeutic procedures.

## Figures and Tables

**Table 1:** Parameters of each power fitted curve related to the experimental measurements.

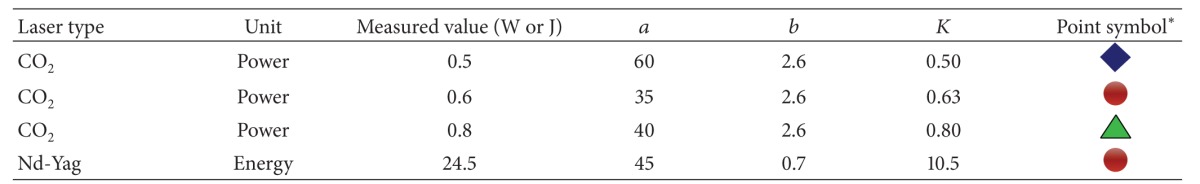

*Point symbols used in Figures 3 and 4.
